# Early structural brain development in infants exposed to HIV and antiretroviral therapy *in utero* in a South African birth cohort

**DOI:** 10.1002/jia2.25863

**Published:** 2022-01-18

**Authors:** Catherine J. Wedderburn, Nynke A. Groenewold, Annerine Roos, Shunmay Yeung, Jean‐Paul Fouche, Andrea M. Rehman, Diana M. Gibb, Katherine L. Narr, Heather J. Zar, Dan J. Stein, Kirsten A. Donald

**Affiliations:** ^1^ Department of Paediatrics and Child Health Red Cross War Memorial Children's Hospital, University of Cape Town Cape Town South Africa; ^2^ Department of Clinical Research London School of Hygiene & Tropical Medicine London UK; ^3^ The Neuroscience Institute University of Cape Town Cape Town South Africa; ^4^ Department of Psychiatry and Mental Health University of Cape Town Cape Town South Africa; ^5^ SA MRC Unit on Risk and Resilience in Mental Disorders, Department of Psychiatry Stellenbosch University Stellenbosch South Africa; ^6^ MRC International Statistics & Epidemiology Group London School of Hygiene & Tropical Medicine London UK; ^7^ MRC Clinical Trials Unit University College London London UK; ^8^ Departments of Neurology, Psychiatry and Biobehavioral Sciences University of California Los Angeles California USA; ^9^ SA MRC Unit on Child & Adolescent Health University of Cape Town Cape Town South Africa; ^10^ SA MRC Unit on Risk and Resilience in Mental Disorders University of Cape Town Cape Town South Africa

**Keywords:** HIV exposure, antiretroviral therapy, magnetic resonance imaging, brain structure, newborn infant, child development

## Abstract

**Introduction:**

There is a growing population of children who are HIV‐exposed and uninfected (HEU) with the successful expansion of antiretroviral therapy (ART) use in pregnancy. Children who are HEU are at risk of delayed neurodevelopment; however, there is limited research on early brain growth and maturation. We aimed to investigate the effects of *in utero* exposure to HIV/ART on brain structure of infants who are HEU compared to HIV‐unexposed (HU).

**Methods:**

Magnetic resonance imaging using a T2‐weighted sequence was undertaken in a subgroup of infants aged 2–6 weeks enrolled in the Drakenstein Child Health Study birth cohort, South Africa, between 2012 and 2015. Mother–child pairs received antenatal and postnatal HIV testing and ART per local guidelines. We compared subcortical and total grey matter volumes between HEU and HU groups using multivariable linear regression adjusting for infant age, sex, intracranial volume and socio‐economic variables. We further assessed associations between brain volumes with maternal CD4 cell count and ART exposure.

**Results:**

One hundred forty‐six infants (40 HEU; 106 HU) with high‐resolution images were included in this analysis (mean age 3 weeks; 50.7% male). All infants who were HEU were exposed to ART (88% maternal triple ART). Infants who were HEU had smaller caudate volumes bilaterally (5.4% reduction, *p* < 0.05) compared to HU infants. There were no group differences in other subcortical volumes (all *p* > 0.2). Total grey matter volume was also reduced in infants who were HEU (2.1% reduction, *p* < 0.05). Exploratory analyses showed that low maternal CD4 cell count (<350 cells/mm^3^) was associated with decreased infant grey matter volumes. There was no relationship between timing of ART exposure and grey matter volumes.

**Conclusions:**

Lower caudate and total grey matter volumes were found in infants who were HEU compared to HU in the first weeks of life, and maternal immunosuppression was associated with reduced volumes. These findings suggest that antenatal HIV exposure may impact early structural brain development and improved antenatal HIV management may have the potential to optimize neurodevelopmental outcomes of children who are HEU.

## 1 INTRODUCTION

Over 1.3 million women living with HIV give birth each year [1, 2]. With effective antiretroviral therapy (ART), the number of infants infected with HIV has reduced to ∼150,000, but paralleled by a growing global population of children who are HIV‐exposed and uninfected (HEU) [2]. Infants who are HEU have been found to have higher mortality and morbidity compared to HIV‐unexposed (HU) infants [3, 4, 5, 6, 7]. Some studies have also suggested that exposure to specific antiretrovirals may be associated with poor pregnancy [6] and neurologic [8, 9] outcomes, while others have not [10, 11]. Further, cohort studies have reported delayed early neurodevelopment in children who are HEU [12, 13], although findings are inconsistent and neurobiological mechanisms are not yet understood [14].

Brain growth and maturation occur rapidly in infancy [15] and form the basis of later cognitive and behavioural outcomes [16]. Advances in neuroimaging allow greater understanding of factors affecting early brain maturation and objective measures of neurodevelopment [17]. While many studies of children with HIV infection have found differences in neuroanatomy [18, 19], magnetic resonance imaging (MRI) studies examining brain development in children who are HEU are scarce. Microstructural differences between children who are HEU and HU have been described using diffusion tensor imaging (DTI) in small sample sizes (HEU group <20) [20, 21, 22, 23]. Yet, despite the potential use of structural MRI, only one retrospective French study has examined brain morphology in young children who were HEU (mean age 26 months) presenting with neurologic symptoms. Of those children, 45% had mitochondrial dysfunction, and 50% of images showed atypical anatomy, including basal ganglia abnormalities and volume loss [24]. However, children in this study were symptomatic at baseline and predominantly exposed to zidovudine; therefore, the findings may not be generalizable.

Sub‐Saharan Africa (SSA) has the highest burden of both HIV and developmental delay globally [1, 25]. Given the relationship between brain structure and function, it is critical to understand the effects of antenatal HIV/ART exposure on brain maturation in this context [17]. The South African Drakenstein Child Health Study (DCHS) birth cohort was established to investigate the early‐life determinants of child health and development [26, 27]. This study is set in an African community with high levels of poverty and other socio‐environmental risk factors [28]. A single report from the DCHS has investigated neonates who are HIV‐exposed using DTI, and found differences in white matter microstructure at this age [21]. Volumetric brain growth in neonates is driven by grey matter development [15]. Prior studies have shown that grey matter may be affected by *in utero factors*, including maternal substance use [29] and child HIV infection [30], yet, to our knowledge, no published studies have examined the effects of antenatal HIV/ART exposure without infection on neonatal grey matter morphometry. Therefore, the aim of this study was to investigate the effects of *in utero* HIV and ART exposure on the neuroanatomy of newborn infants who are HEU compared to HU. We hypothesized that infants who are HEU would have altered grey matter volumes compared to HU infants.

## 2 METHODS

### 2.1 Participants

We conducted a prospective neuroimaging study of neonates nested within the DCHS, an observational population‐based birth cohort in the Western Cape of South Africa [26, 27]. Pregnant women were recruited into the DCHS from two public sector primary healthcare clinics, Mbekweni (serving a predominantly black African community) and TC Newman (serving a mixed ancestry community). Mothers were enrolled into the DCHS during their second trimester if they were at least 18 years old, between 20 and 28 weeks’ gestation and intended to remain in the area [26, 27].

**Figure 1:**
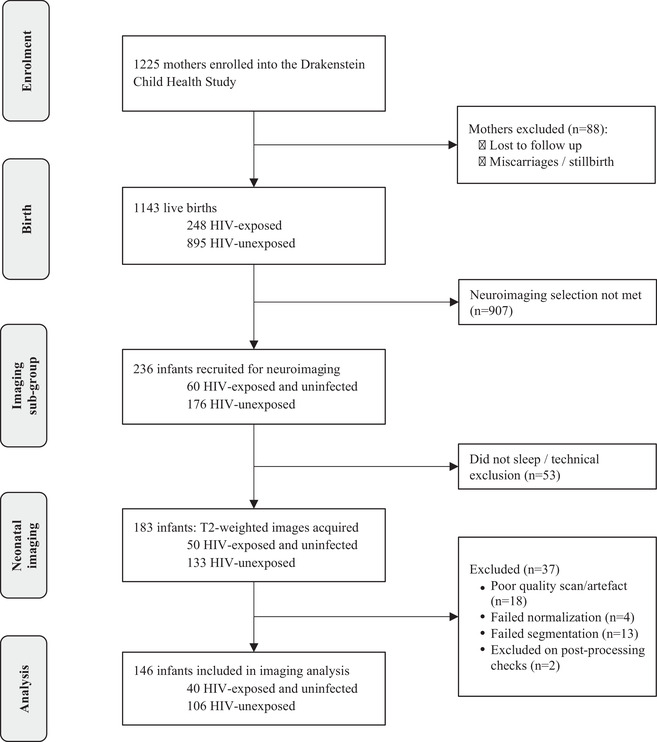
Drakenstein Child Health Study cohort flow chart of children with neuroimaging.

Between 2012 and 2015, 1143 infants were born to 1137 mothers (Figure 1). Antenatal maternal HIV prevalence was 21%, but only two infants were HIV infected [28]. After birth, a sub‐group of 236 newborn infants were invited for neuroimaging (20.6%) [31]. Infants were included if they were aged between 2 and 6 weeks and excluded if they had: (1) medical comorbidities, including a genetic syndrome, neurological disorder or congenital abnormality; (2) history of prematurity (gestation <36 weeks); (3) low Apgar score (<7 at 5 minutes); (4) neonatal intensive care admission; (5) maternal use of illicit drugs during pregnancy; (6) MRI contraindications; or (7) infant HIV infection [29]. Convenience sampling was used to select from eligible infants. In this high‐risk population, we paid particular attention to other known risk factors associated with adverse neurodevelopment, including maternal depression and alcohol use, and account for these in sensitivity analyses.

### 2.2 Ethical considerations

The study was approved by the Faculty of Health Sciences, Human Research Ethics Committee, University of Cape Town (401/2009, 525/2012 and 044/2017) and the Western Cape Department of Provincial Health Research Committee. Written informed consent by mothers was given at enrolment and at the start of each neuroimaging session.

### 2.3 Procedures

#### 2.3.1 HIV/ART data

All women underwent routine HIV testing in pregnancy as per the Western Cape prevention of mother‐to‐child transmission of HIV (PMTCT) guidelines [32, 33]. Mothers who tested negative were retested throughout pregnancy and the postpartum period every 3 months to detect seroconversion. Mothers living with HIV were enrolled into the Provincial PMTCT programme and initiated on ART per guidelines at the time. Before May 2013, the first‐line regimen depended on maternal clinical and immunological status consisting of triple ART or zidovudine from 14 weeks’ gestation; from May 2013, universal triple ART (commonly tenofovir, emtricitabine plus efavirenz) was given to all pregnant women for life. Infants who were HEU received prophylaxis of nevirapine with or without zidovudine. Additional data on maternal CD4 and viral load during pregnancy were obtained using the online National Health Laboratory Service. HIV‐exposed infants were tested for HIV using PCR at 6 weeks of age per the guidelines; all HEU infants were confirmed to have a negative HIV test result. HU infants were born to mothers with confirmed HIV negative status.

#### 2.3.2 Socio‐demographic variables

Socio‐demographic data were collected using validated questionnaires during interviews with trained study staff [34]. Birth data were abstracted from hospital records. Child gestational age at delivery was calculated to define prematurity based on antenatal ultrasound if available, otherwise using symphysis‐fundal height or maternal report of last menstrual period [28]. Maternal data on alcohol use, smoking and depression were collected at antenatal visits and birth [27].

#### 2.3.3 Cytomegalovirus testing

Nasopharyngeal swabs were collected from a subgroup of infants (*n* = 111) between birth and the MRI scan as part of the DCHS protocol. Cytomegalovirus (CMV) DNA was measured using quantitative multiplex real‐time PCR on FTDResp33 (Fast‐track Diagnostics, Luxembourg). These methods are described in full elsewhere [35].

#### 2.3.4 Neuroimaging acquisition

T2‐weighted MR images [36] were acquired at the Cape Universities Brain Imaging Centre, at Tygerberg Hospital from September 2012 to September 2015 using a Siemens Magnetom 3T Allegra MRI scanner (Erlangen, Germany). Neuroimaging was conducted during natural sleep without sedation [21] (Supporting Information).

#### 2.3.5 Neuroimaging processing

Neuroimaging data were available for 183 neonates (50 HEU; 133 HU). T2‐weighted images were processed using Statistical Parametric Mapping (SPM8) software blinded to child HIV/ART exposure status, to generate measures of brain structure. Images were segmented and total grey matter and subcortical regions‐of‐interest (thalamus, caudate, putamen, pallidum, amygdala and hippocampus) were defined per the automated anatomical labelling atlas [37] adapted to a neonate grey matter template in Montreal Neurological Institute standard space [38]. Volumes were extracted from segmented grey matter images bilaterally. Quality control was performed by two researchers to identify any scan artefacts and errors of segmentation. Outlier detection was performed using the ENIGMA protocol [39]. Any regions marked as a statistical outlier were re‐inspected to confirm accurate segmentation.

The mean of the left and right hemispheres was calculated for each subcortical structure. Intracranial volume (ICV) was calculated as total grey matter, white matter and cerebrospinal fluid.

### 2.4 Statistical analysis

Socio‐demographic and clinical characteristics were compared between HEU and HU groups using unpaired *t*‐tests or Mann–Whitney U tests (continuous variables), and chi‐squared tests or Fisher's exact tests (categorical variables). Group differences in subcortical and total grey matter volumes were assessed using linear regression. In each model, HIV exposure was considered as the independent variable and grey matter volume as the dependent variable. Confounders were selected *a priori*. Minimally adjusted models included infant age at scan, infant sex and ICV (to account for variability in head size). Fully adjusted models were created using a Directed Acyclic Graph [4, 12] to determine the minimal adjustment set of variables and additionally included maternal education, household income and maternal age. Standardized effect sizes were calculated using Cohen's d. Residuals were checked for normality using quantile–quantile plots and homogeneity of variance using scatterplots. Percentage differences (% absolute difference in volume between HEU and HU infants relative to the mean volume in the control group for the particular structure) were also calculated.

Among infants who were HEU, the effects of maternal disease severity (measured through CD4 cell count) and maternal ART were explored through secondary regression analyses. CD4 cell counts were binned into three categories, <350, 350–500 and >500, per previous guidelines [33]. For ART exposure, analyses were performed firstly comparing infants exposed to maternal triple ART versus zidovudine only; and secondly, examining timing of initial ART exposure, comparing maternal ART initiation *preconception* versus *during pregnancy* given reports of adverse outcomes associated with ART exposure at conception [40]. We also conducted analyses limiting to infants exposed to the same WHO‐recommended first‐line regimen that most women received (tenofovir + emtricitabine/lamivudine + efavirenz) to allow us to examine the timing of initial ART exposure without the confounding effects of different antiretroviral drugs.

Finally, sensitivity analyses were performed to assess the robustness of the results by: (1) excluding imaging outliers; (2) restricting to neonates ≤28 days old at imaging; (3) restricting to infants from one clinic, given site differences and that site was closely correlated to HIV status; (4) assessing additional potential confounders, including maternal prenatal alcohol use, smoking and depression, that may be on the causal pathway; and (5) excluding infants who tested CMV positive at any point (Supporting Information).

Data analysis was performed using STATA (StataCorp Inc, College Station, TX, USA) version 14.2. *p* < 0.05 was used as the threshold of statistical significance. The mean volume of each bilateral subcortical structure was used to minimize the number of comparisons; however, where a region was associated with HIV exposure status (*p* < 0.05), we conducted exploratory analyses stratified by hemisphere. We also performed additional correction for multiple comparisons across the subcortical regional models (*n* = 6) using the standard false discovery rate method with a false‐positive rate of 5% (*q* = 0.05) [41].

## 3 RESULTS

### 3.1 Characteristics of infants who are HEU and HU

A total of 146 infants (40 HEU; 106 HU) had T2‐weighted structural MRIs that passed quality control (Figure 1). HEU and HU groups were comparable in terms of age at scan, sex and socio‐economic factors, although mothers with HIV were older. Median age at scanning was 21 days (IQR 18–26) and 51% were male. Higher prevalence of prenatal smoking, alcohol use and depression occurred in the HU group (Table 1). All infants who were HEU were ART exposed (88% to maternal triple ART; 38% exposed from conception). Median maternal CD4 cell count during pregnancy was 423 cells/mm^3^ (IQR 286–594). The imaging subsample was largely representative of the population included in the wider study (Table S1).

**Table 1:** Socio‐demographic and clinical characteristics of infants according to HIV exposure

Variable	Total (*n* = 146)	HEU (*n* = 40)	HU (*n* = 106)	*p*‐value
*Socio‐demographic characteristics*				
Child age at scan, days	21 (18–26)	23 (19–28)	21 (18–26)	0.326
Child post‐conceptual age at scan, days	298 (291–307)	298 (286–309)	298 (293–306)	0.714
Sex				
Female	72 (49.3%)	18 (45.0%)	54 (50.9%)	0.522
Male	74 (50.7%)	22 (55.0%)	52 (49.1%)	
Site				
TC Newman	73 (50.0%)	1 (2.5%)	72 (67.9%)	<0.001*
Mbekweni	73 (50.0%)	39 (97.5%)	34 (32.1%)	
Monthly household income (ZAR)				
<R1000 (<∼$75)	49 (33.6%)	13 (32.5%)	36 (34.0%)	0.513
R1000–R5000 (∼$75–375)	75 (51.4%)	23 (57.5%)	52 (49.1%)	
>R5000 (>∼$375)	22 (15.1%)	4 (10.0%)	18 (17.0%)	
Maternal education				
Any primary	7 (4.8%)	2 (5.0%)	5 (4.7%)	0.915
Any secondary	73 (50.0%)	20 (50.0%)	53 (50.0%)	
Completed secondary	58 (39.7%)	15 (37.5%)	43 (40.6%)	
Any tertiary	8 (5.5%)	3 (7.5%)	5 (4.7%)	
Maternal employment status (employed)	40 (27.4%)	11 (27.5%)	29 (27.4%)	0.986
Relationship status (married/cohabitating)	63 (43.5%)	16 (40.0%)	47 (44.8%)	0.605
Maternal age at birth, years	26.9 (22.0–31.6)	29.4 (24.6–33.2)	26.1 (21.7–30.0)	0.017*
Gestational age at birth, weeks	39 (38–40)	39 (38–40)	39 (38–40)	0.390
Birthweight, g	3157 (462)	3110 (443)	3174 (469)	0.462
Birth head circumference, cm	33.8 (1.7)	33.8 (1.8)	33.8 (1.7)	0.957
Maternal smoking during pregnancy				
Active	45 (31.0%)	6 (15.0%)	39 (37.1%)	
Passive	61 (42.1%)	22 (55.0%)	39 (37.1%)	
Non‐smoker	39 (26.9%)	12 (30.0%)	27 (25.7%)	0.031*
Maternal alcohol use during pregnancy	25 (17.9%)	2 (5.6%)	23 (22.1%)	0.025*
Maternal depression	44 (31.4%)	6 (16.7%)	38 (36.5%)	0.027*
Maternal hospitalization	8 (5.5%)	1 (2.5%)	7 (6.6.%)	0.446
*Neuroanatomical variables*				
Total intracranial volume (mm^3^)	425,903 (4434)	425,352 (5237)	426,111 (4100)	0.674
*Maternal and child HIV variables*				
Maternal HIV diagnosis time‐point				
Before pregnancy		27 (71.1%)		
During pregnancy		11 (29.0%)		
Maternal CD4 cell count				
Median (interquartile range) (cells/mm^3^)		423 (286–594)		
<350 cells/mm^3^		13 (39.4%)		
350–500 cells/mm^3^		8 (24.2%)		
>500 cells/mm^3^		12 (36.4%)		
Maternal viral load (VL) in pregnancy				
Lower than detectable limit (<40 copies/ml)		19 (73.1%)		
VL detectable (≥40–1000 copies/ml)		5 (19.2%)		
Virally unsuppressed (≥1000 copies/ml)		2 (7.7%)		
Antiretroviral drug initiation				
Before conception		15 (37.5%)		
During pregnancy		25 (62.5%)		
Antiretroviral regimen during pregnancy				
Monotherapy with AZT (zidovudine)		5 (12.5%)		
2 NRTIs + NNRTI (first line)		34 (85.0%)		
2 NRTIs + PI (second line)		1 (2.5%)		
Infant prophylaxis				
NVP [nevirapine] alone		35 (87.5%)		
NVP + AZT		5 (12.5%)		

Note: Data are presented as *n*/*N* (%), mean (SD) or median (IQR). *p*‐values represent group‐comparison with two‐sided unpaired *t*‐tests or Mann–Whitney U tests (continuous variables), or chi‐squared or Fisher's exact test if any *n* <5 (categorical variables) as appropriate.

Abbreviations: AZT, zidovudine; HEU, HIV‐exposed and uninfected; HU, HIV‐unexposed; NNRTI, non‐nucleoside reverse transcriptase inhibitor; NRTI, nucleoside reverse transcriptase inhibitor; NVP, nevirapine; PI, protease inhibitor; VL, viral load.

* *p* < 0.05. Percentages are cited among those with non‐missing values. Missing data: relationship status (*n* = 1); birthweight (*n* = 1); smoking (*n* = 1); alcohol (*n* = 6); depression (*n* = 6); HIV diagnosis time‐point (*n* = 2); CD4 (*n* = 7); and viral load (*n* = 14). Specific variables were assessed as follows: (1) maternal smoking was measured by urine cotinine levels taken antenatally/birth; (2) maternal alcohol use was assessed and quantified using the Alcohol, Smoking and Substance Involvement Screening Test (ASSIST) and retrospectively collected data on moderate‐severe alcohol use in pregnancy forming a dichotomous measure; (3) maternal depression in pregnancy was measured using the Edinburgh postnatal depression scale (EPDS); (4) the lowest maternal CD4 during pregnancy was used to reflect maternal immunosuppression in pregnancy; (5) maternal viral load was categorized into <40 copies/ml as lower than the detectable limit, ≥40 to <1000 copies/ml as detectable and ≥1000 copies/ml as unsuppressed. Where there was more than one result, the highest viral load during pregnancy was taken; (6) of those mothers initiating ART during pregnancy, timing of initiation: 6 (24%) first trimester, 14 (56%) second/third trimester, 5 (20%) unknown; (7) first‐line triple therapy: a non‐nucleoside reverse‐transcriptase inhibitor backbone and two nucleoside reverse transcriptase inhibitors, most commonly efavirenz with tenofovir and emtricitabine as a fixed‐dose combination (*n* = 31, 77.5%); however, some mothers received nevirapine‐based treatment (*n* = 3).

### 3.2 Subcortical volumes

**Figure 2:**
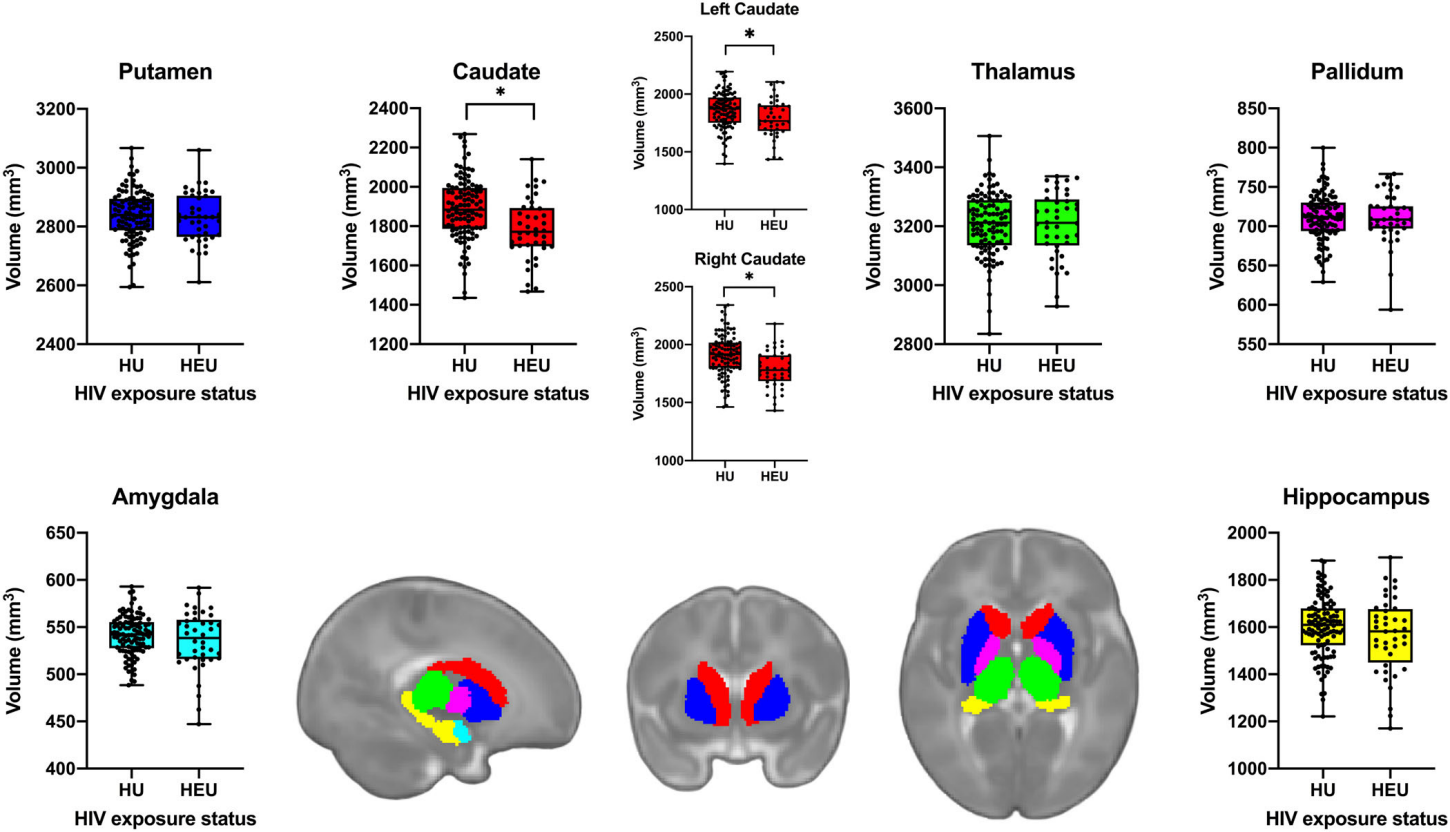
Comparison of subcortical volumes between infants who are HEU and HU. Mean volumes (of left and right hemispheres) for each subcortical region comparing infants who are HEU (*n* = 40) and HU (*n* = 106) with an associated atlas of brain regions. *p < 0.05 after multiple comparison correction. The box‐and‐whisker plots demonstrate the data distribution and group differences of the mean volume of left and right hemispheres in mm^3^. In all plots, the middle line represents the median, and the upper and lower bounds of the boxes are the first and third quartiles, respectively. The whiskers extend from highest to lowest data point, and all data points are plotted. Representative atlas images are in sagittal, coronal and axial views (from left to right).

Infants who were HEU had smaller caudate volumes in comparison to HU (1785 vs. 1886 mm^3^, *p* = 0.0009, Cohen's d effect size −0.58 [95% CI −0.95 to −0.21]) independent of infant age, sex and ICV (Table 2 and Figure 2). When adjusting for socio‐economic confounders (maternal age, education and household income), the associations held with a similar effect size −0.57 (−0.94 to −0.20) and remained significant after multiple comparison correction (Table 2). This effect was evident bilaterally (left hemisphere *p* = 0.015, effect size: −0.45 [−0.82 to −0.09]; right hemisphere *p* = 0.0002, effect size: −0.62 [−1.00 to −0.25]) with volumetric reductions of 5.4% overall, 4.1% left hemisphere and 6.6% right hemisphere. There were no group differences in other subcortical regions (all *p* > 0.2).

**Table 2:** Adjusted mean differences in total and regional grey matter volumes between infants who are HIV‐exposed and uninfected compared to HIV‐unexposed

Brain volumes	HEU Mean (SD) (*n* = 40)	HU Mean (SD) (*n* = 106)	Minimally adjusted^a^ difference (95% CI)	*p*‐value	Effect size, Cohen's d (95% CI)	Fully adjusted^b^ difference (95% CI)	*p*‐value	Effect size, Cohen's d (95% CI)
*Global*								
Total grey matter	233,713 (15,402)	238,676 (12,127)	−4385 (−8455 to −315)	0.035*	−0.33 (−0.69 to 0.04)	−4352 (−8525 to −179)	0.041*	−0.33 (−0.69 to 0.04)
*Subcortical regions*								
Thalamus	3205 (113)	3209 (105)	3.76 (−34.03 to 41.54)	0.845	0.04 (−0.33 to 0.40)	6.27 (−32.50 to 45.04)	0.750	0.06 (−0.31 to 0.42)
Caudate	1785 (155)	1886 (158)	−97.54 (−154.39 to −40.68)	<0.001* ^,^ ^§^	−0.58 (−0.95 to −0.21)	−96.67 (−154.33 to −39.00)	0.001* ^,^ ^§^	−0.57 (−0.94 to −0.20)
Putamen	2830 (86)	2839 (85)	−5.60 (−35.57 to 24.36)	0.712	−0.07 (−0.43 to 0.30)	−3.91 (−34.85 to 27.03)	0.803	−0.05 (−0.41 to 0.32)
Pallidum	711 (33)	712 (30)	−0.32 (−11.15 to 10.50)	0.953	−0.01 (−0.37 to 0.35)	−0.43 (−11.62 to 10.76)	0.940	−0.01 (−0.38 to 0.35)
Hippocampus	1563 (162)	1601 (128)	−24.42 (−68.40 to 19.56)	0.274	−0.18 (−0.54 to 0.19)	−25.28 (−70.64 to 20.07)	0.272	−0.18 (−0.55 to 0.18)
Amygdala	535 (32)	541 (21)	−4.05 (−12.14 to 4.04)	0.324	−0.16 (−0.53 to 0.20)	−3.76 (−12.16 to 4.63)	0.377	−0.15 (−0.52 to 0.21)

Note: Subcortical volumes (mean of left and right hemispheres) in mm^3^, mean differences (regression coefficients minimally and fully adjusted in multiple regression models), *p* values and effect sizes for associations between brain volumes and HIV exposure. Effect sizes were calculated using Cohen's d with associated 95% confidence intervals. Residuals were assessed for each model using quantile–quantile plots and were normally distributed. Linear regression models shown where negative estimates indicate that HIV exposure is associated with lower volumes in that region.

Abbreviations: CI, confidence interval; HEU, HIV‐exposed and uninfected; HU, HIV‐unexposed.

^a^ Minimally adjusted models included child age at scan, child sex and intracranial volume.

^b^ Fully adjusted models included child age at scan, child sex, intracranial volume, maternal education, household income and maternal age.

* Uncorrected *p* < 0·05.

^§^ *p*‐Values survive multiple comparison correction using the false discovery rate across the six subcortical regions, which generated a corrected overall *p*‐value of 0.0083.

For regions with a significant difference between HEU and HU infants, the percentage volume difference was calculated as the absolute mean difference expressed as a percentage of the mean volume of the HU control group: total grey matter = 2.1% reduction; total caudate = 5.4% reduction. Post hoc the caudate was assessed bilaterally: left hemisphere: 4.1% reduction, fully adjusted Cohen's d effect size: −0.45 (95% CI −0.82 to −0.09). Right hemisphere: 6.6% reduction, fully adjusted Cohen's d effect size: −0.62 (95% CI −1.00 to −0.25).

### 3.3 Total grey matter

**Figure 3:**
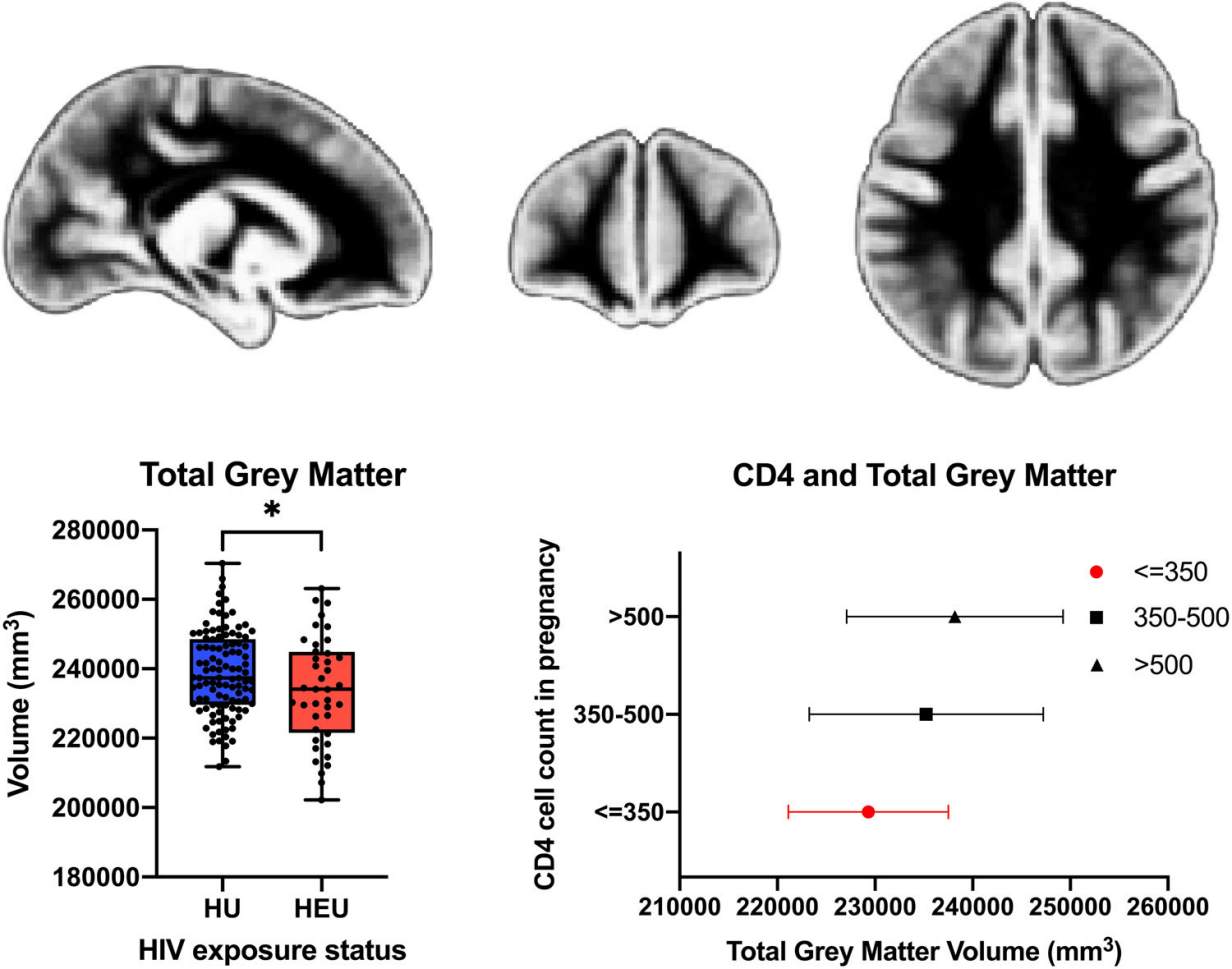
Assessment of the impact of HIV/ART exposure on total grey matter and associations with maternal immune status. (a) Comparison of total grey matter volume between infants who are HEU and HU. Total grey matter volumes are plotted comparing infants who are HEU (*n* = 40) and HU (*n* = 106) with an associated neonatal atlas of grey matter. *p < 0.05. We present box‐and‐whisker plots to demonstrate the data distribution. In all plots, the middle line represents the median, and the upper and lower bounds of the boxes are the first and third quartiles, respectively. The whiskers extend from highest to lowest data point, and all data points are plotted. (b) Association of maternal CD4 cell count with total grey matter volume in HEU infants. Mean volumes for children born to mothers with CD4 > 500 cells/mm^3^ (*n* = 12); CD4 350–500 cells/mm^3^ (*n* = 8); and CD4 < 350 cells/mm^3^ (*n* = 13).

Total grey matter volume was 2.1% smaller in infants who were HEU (233,713 vs. 238,676 mm^3^, *p* = 0.035, minimally adjusted Cohen's d effect size: −0.33 [−0.69 to 0.04]) compared to HU infants (Table 2 and Figure 3). There remained strong evidence for association after further adjusting for potential socio‐economic confounders (Table 2).

### 3.4 Sensitivity analyses

We confirmed the findings by conducting the analyses excluding statistical outliers (Table S2) and restricting firstly to those infants who were ≤28 days of age (Table S3); and secondly to those infants who were attending Mbekweni clinic, where the majority of infants who are HEU were attending (Table S4), resulting in associations of a similar magnitude and direction. Our findings persisted after adjusting for relevant psychosocial variables that differed between groups, including prenatal exposure to maternal depression, smoking and alcohol that were higher in the control HU group (Tables S5–S7). No child had evidence of congenital CMV disease. A low prevalence of infant CMV infection was detected on nasopharyngeal swabs taken prior to the scan date, with one HEU and one HU infant testing positive (no group difference, *p* = 0.570). Excluding these infants from the analysis did not meaningfully change the associations or effect sizes (Table S8).

### 3.5 Association with maternal immune function and ART

In exploratory analyses, we found maternal CD4 predicted infant total grey matter volumes (Figure 3 and Table S9). Our results suggest that there is a dose–response association with lower maternal CD4 associated with smaller infant volumes, and infants who were HEU born to mothers with CD4 cell count <350 cells/mm^3^ had the lowest volumes. We found no relationship between maternal ART regimen or timing of initiation and infant global and regional brain volumes (*p* > 0.05) (Table S10). In analyses restricted to infants who were HEU exposed to the same first‐line triple ART, there was also no effect of ART initiation timing. In this sub‐group, the relationship between maternal immunological compromise and decreased infant grey matter volumes was strengthened for both caudate and total grey matter (Table 3).

**Table 3:** Impact of maternal ART timing of initiation and maternal CD4 count during pregnancy on caudate and total grey matter volumes restricted to HIV‐exposed and uninfected infants born to mothers on first‐line triple ART

	Caudate		Total grey matter	
	Adjusted coefficient^a^ (95% CI)	*p*‐Value	Adjusted coefficient^a^ (95% CI)	*p*‐Value
*ART initiation*				
Before conception	*Reference*		*Reference*	
During pregnancy	−58.30 (−164.40 to 47.81)	0.263	−71.68 (−11,225 to 11,081)	0.989
*CD4*				
CD4 >500	*Reference*		*Reference*	
CD4 350–500	−25.52 (−150.70 to 99.67)	0.674	−5229 (−18,387 to 7930)	0.415
CD4 <350	−128.72 (−248.56 to −8.88)	0.037^*^	−15,023 (−27,619 to −2426)	0.022^*^

Note: Mean differences (adjusted regression coefficients with 95% CIs) and *p*‐values for associations between brain volumes and maternal ART initiation timing in relation to pregnancy and CD4 cell count, restricted to those mothers on the same first‐line ART regimen consisting of tenofovir + emtricitabine/lamivudine + efavirenz. ART timing was dichotomized into initiation before or during pregnancy. Total N = 25 (ART initiation before conception [n = 7], during pregnancy [n = 18]; CD4 >500 [n = 7], 350–500 [n = 6], <350 [n = 12]).

Abbreviations: ART, antiretroviral therapy; CI, confidence interval.

^a^ Models were adjusted to include maternal CD4 cell count, ART initiation timing, child age at scan, child sex and intracranial volume (only ART initiation timing and CD4 are shown).

* *p* < 0·05.

Post hoc the caudate was assessed bilaterally for the effect of CD4<350: left hemisphere: CD4 <350 adjusted coefficient: −131.41 (−260.99 to −1.84) *p* = 0.047; right hemisphere: CD4<350 adjusted coefficient: −126.03 (−258.30 to 6.24), *p* = 0.061.

## 4 DISCUSSION

In this study, we used MRI to examine the impact of HIV/ART exposure on brain structure. Overall, infants who were HEU had smaller total grey matter and subcortical caudate volumes bilaterally compared to infants who were HU, independent of ICV and socio‐economic factors. In exploratory analyses, maternal immunosuppression was associated with reduced volumes. Our results suggest that *in utero* exposure to HIV may affect brain maturation of specific regions, and changes in brain structure in infants who are HEU can be detected at a very early stage of neurodevelopment. This concurs with recent literature on early growth trajectories, suggesting prenatal origins of developmental abnormalities in children who are HEU [42, 43].

We found that the caudate, a major nucleus of the basal ganglia and integral part of the cortico‐striatal network, was smaller in volume in infants who were HEU compared to HU. Other subcortical regions were not affected. Although there are no prior publications on basal ganglia volume in children who are HEU, basal ganglia abnormalities have been frequently reported in adults [44] and children with HIV [19, 30], despite ART. Furthermore, magnetic resonance spectroscopy studies have found altered basal ganglia metabolite levels in school‐aged children who are HEU at 9 years [45] (although not 11 years [46]), and who are living with HIV [47], suggesting that basal ganglia neurons may be particularly susceptible to the effects of HIV exposure [45]. The close proximity of the caudate to the cerebrospinal fluid [44] may also make it more vulnerable to changes to the *in utero* environment.

Our results also show that HIV exposure was associated with lower total grey matter volume, which builds on evidence of volume reductions reported in a small study of children aged 2 years [24]. However, this was a French retrospective study of children presenting with neurological symptoms, and the generalizability of these findings to the large HEU population in SSA is uncertain. Studies in children with HIV infection [30], adolescents [48] and adults [44] on ART have also reported smaller global grey matter volumes. Although mechanisms are likely to differ between children living with HIV and HEU, our data suggest that there may be similarities across neurobiological pathways for the impact of HIV on the developing brain in the context of exposure and infection; or the downstream effects of altered immune function in pregnancy may have a similar impact.

Various potential mechanisms are hypothesized by which maternal HIV exposure may impact a child's developing nervous system. In exploratory analyses, we found an association between maternal CD4 cell count during pregnancy and total grey matter and caudate volumes. Immune activation in pregnancy has been implicated in various neuropsychiatric disorders in the offspring [49], and associated with immunological dysregulation in children who are HEU [50] and altered brain development [50, 52]. Importantly, the identified dose–response association between grey matter volume and maternal CD4 suggests that close maternal HIV management during pregnancy, including viral load monitoring and patient education to prevent immune compromise, may be key for promoting optimal fetal neural development.

Previous work has suggested potential neurotoxic effects of ART on the developing brain [24, 53] and that timing of exposure may be a determinant of outcomes [40]. We investigated maternal ART regimen and timing of initial exposure in relation to conception. We found no relationship between ART exposure and brain volumes in this cohort. However, most mothers were on first‐line triple therapy limiting statistical power to separate the effects of HIV and ART. In analyses restricted to HEU infants exposed to the same first‐line ART, removing the potential diluting effect of different antiretrovirals, there was again no effect of ART initiation timing. Further work is needed in this area, particularly given the previously described associations between efavirenz and neurobehaviour [40, 53] and the new WHO guidelines recommending dolutegravir‐based ART [54]. While dolutegravir has improved efficacy [55], there were concerns raised in 2018 related to an increased risk of neural tube defects in women conceiving on dolutegravir [56, 57]; however, recent data show that differences between dolutegravir‐ and non‐dolutegravir regimens are no longer significant [10, 58]. Long‐term safety information on integrase strand transfer inhibitors is needed, and monitoring of child brain outcomes is critical.

Grey matter growth is fundamental for cognitive, language and motor skill development [15], and the caudate plays a role in motivation, movement, cognition and verbal fluency [59], as such, these structural deficiencies may have important neurodevelopmental implications. Postnatal maturation of the brain grey matter and subcortical structures occurs most rapidly in early infancy [15, 17], and evidence from studies of premature infants has shown that deep grey brain volumes, particularly the caudate, predict academic outcomes in childhood [60, 61]. Previous cohort studies have reported delayed neurocognitive development in children who are HEU compared to HU [12, 62], although findings are inconsistent [14]. Delineating any differences in brain anatomy will help further understand the neurodevelopmental trajectories of children who are HEU. Our findings provide evidence for subtle neuroanatomical differences between infants who are HEU versus HU early in life. Future research is needed to further explore underlying causal pathways and the complex interactions between factors that impact brain maturation, including the role of biological, environmental and psychosocial variables [4].

Strengths of the study include a well‐characterized cohort and an HEU group representative of other HEU populations across South Africa [63]. We present, to our knowledge, the largest neuroimaging study of children who are HEU during a period of importance for structural and functional development [17] that allows better differentiation between *in utero* versus postnatal risk factors than studies of older children. We also considered the potential impact of infant CMV infection. However, some important limitations of our study require consideration. Our sample size limited statistical power in the exploratory CD4 and ART analyses, and we were unable to examine maternal viral load. Although we excluded infants with known risk factors for neurodevelopment and adjusted for other covariates, we acknowledge the potential for residual confounding. More studies are needed to replicate these findings and assess generalizability. Finally, we only report a single imaging time‐point and note the age of the cohort (2012–2015) as a limitation. Although overall PMTCT coverage at the time of this study was similar to many SSA countries currently [2], our cohort is pre‐dolutegravir. The clinical relevance and longitudinal trajectories of the identified volumetric abnormalities need to be confirmed in future prospective studies, along with investigation of new ART regimens.

## 5 CONCLUSIONS

We show that infants who are HEU had smaller caudate and total grey matter volumes compared to HU infants. Scans were conducted at a median of 21 days of life, limiting exposure to postnatal factors that may also influence brain development. The results are further strengthened by robustness after accounting for potential socio‐economic confounders, and associations with maternal immune status. These findings suggest that antenatal HIV exposure may impact early brain structural development and maturation, and demonstrate that neuroanatomical changes are present soon after birth. This has important implications for understanding the risk of long‐term neurodevelopmental impairment in the growing HEU population and suggests that improved HIV prevention, along with better maternal HIV management in pregnancy, may have the potential to optimize child brain development. Longitudinal follow up is needed to determine underlying mechanisms and whether these abnormalities persist into later childhood.

## COMPETING INTERESTS

The authors declare no competing interests.

## AUTHORS’ CONTRIBUTIONS

CJW processed the imaging data with supervision from NAG, and was responsible for statistical analysis, interpretation of results and drafting of the manuscript. KAD was responsible for the neuroimaging and with NAG, SY and DMG assisted with conception and manuscript revisions. AR assisted with data collection, AMR supported the data analysis, JPF and KLN provided imaging advice and all gave input into the manuscript. HJZ devised the Drakenstein Child Health study, DJS conceived the neuroimaging and maternal aspects, and both revised the manuscript critically for intellectual content. All authors have read and approved the final manuscript.

## FUNDING

The DCHS study is funded by the Bill & Melinda Gates Foundation [OPP 1017641]. Additional support for HJZ, DJS and KAD was provided by the South African Medical Research Council. CJW was supported by the Wellcome Trust through a Research Training Fellowship [203525/Z/16/Z]. NAG was supported by a Claude Leon Postdoctoral Fellowship. Support for the neuroimaging was also received by KAD from an ABMRF young investigator grant, the Brain & Behavior Research Foundation Independent Investigator grant (24467) and NIH‐R21AA023887 and the Harry Crossley Foundation. AMR is additionally supported by the UK Medical Research Council (MRC) and the UK Department for International Development (DFID) under the MRC/DFID Concordat agreement, which is also part of the EDCTP2 programme supported by the European Union grant reference (MR/R010161/1). The funders had no role in the study design, data collection, analysis or interpretation, report writing or decision to submit for publication. The corresponding author had full access to study data and final responsibility for the decision to submit for publication.

## Supporting information

Supporting Information
**Table S1**. Sociodemographic characteristics of children with neuroimaging versus those without in the Drakenstein Child Health Study
**Table S2**. Adjusted mean differences in grey matter volumes according to HIV exposure status excluding statistical outliers
**Table S3**. Adjusted mean differences in grey matter volumes according to HIV exposure status restricted to children ≤28 days
**Table S4**. Adjusted mean differences in grey matter volumes according to HIV exposure status restricted to one clinic
**Table S5**. Grey matter volumes according to HIV exposure status assessing the effect of maternal depression on the exposure‐outcome relationship
**Table S6**. Grey matter volumes according to HIV exposure status assessing the effect of smoking on the exposure‐outcome relationship.
**Table S7**. Grey matter volumes according to HIV exposure assessing the effect of alcohol on the exposure‐outcome relationship
**Table S8**. Adjusted mean differences in grey matter volumes according to HIV exposure status excluding CMV positive cases
**Table S9**. Impact of maternal HIV disease severity (immunological compromise) on caudate and total grey matter volumes
**Table S10**. Impact of maternal ART regimen and timing of initiation on caudate and total grey matter volumes

## Data Availability

The de‐identified data that support the findings of this study are available from the authors upon reasonable request as per DCHS cohort guidelines.
