# Supplementary material for: Early structural brain development in infants exposed to HIV and antiretroviral therapy in utero in a South African birth cohort
Source: J Int AIDS Soc. 2022 Jan 18;25(1):e25863. doi: 10.1002/jia2.25863 (PMC8765561; doi:10.1002/jia2.25863)
Supplement: Supplementary file 1 — Supporting Information Table S1. Sociodemographic characteristics of children with neuroimaging versus those without in the Drakenstein Child Health Study Table S2. Adjusted mean differences in grey matter volumes according to HIV exposure status excluding statistical outliers Table S3. Adjusted mean differences in grey matter volumes according to HIV exposure status restricted to children ≤28 days Table S4. Adjusted mean differences in grey matter volumes according to HIV exposure status restricted to one clinic Table S5. Grey matter volumes according to HIV exposure status assessing the effect of maternal depression on the exposure‐outcome relationship Table S6. Grey matter volumes according to HIV exposure status assessing the effect of smoking on the exposure‐outcome relationship. Table S7. Grey matter volumes according to HIV exposure assessing the effect of alcohol on the exposure‐outcome relationship Table S8. Adjusted mean differences in grey matter volumes according to HIV exposure status excluding CMV positive cases Table S9. Impact of maternal HIV disease severity (immunological compromise) on caudate and total grey matter volumes Table S10. Impact of maternal ART regimen and timing of initiation on caudate and total grey matter volumes [file JIA2-25-e25863-s001.pdf]

Supplementary Information

Early structural brain development in infants exposed to HIV and antiretroviral therapy *in utero* in a South African birth cohort

Catherine J Wedderburn, Nynke A Groenewold, Annerine Roos, Shunmay Yeung, Jean-Paul Fouche, Andrea M Rehman, Diana M Gibb, Katherine L Narr, Heather J Zar, Dan J Stein, Kirsten A Donald

Correspondence to: catherine.wedderburn@uct.ac.za

Supplementary Methods: Neuroimaging acquisition, processing, data quality assessment and analysis

Supplementary Table S1: Sociodemographic characteristics of children with neuroimaging versus those without in the Drakenstein Child Health Study

Supplementary Table S2: Adjusted mean differences in grey matter volumes according to HIV exposure status excluding statistical outliers

Supplementary Table S3: Adjusted mean differences in grey matter volumes according to HIV exposure status restricted to children ≤28 days

Supplementary Table S4: Adjusted mean differences in grey matter volumes according to HIV exposure status restricted to one clinic

Supplementary Table S5: Grey matter volumes according to HIV exposure status assessing the effect of maternal depression on the exposure-outcome relationship

Supplementary Table S6: Grey matter volumes according to HIV exposure status assessing the effect of smoking on the exposure-outcome relationship.

Supplementary Table S7: Grey matter volumes according to HIV exposure assessing the effect of alcohol on the exposure-outcome relationship

Supplementary Table S8: Adjusted mean differences in grey matter volumes according to HIV exposure status excluding CMV positive cases

Supplementary Table S9: Impact of maternal HIV disease severity (immunological compromise) on caudate and total grey matter volumes

Supplementary Table S10: Impact of maternal ART regimen and timing of initiation on caudate and total grey matter volumes

Supplementary References

Supplementary Methods

Neuroimaging acquisition details

3D T2-weighted MR images were acquired at the Cape Universities Brain Imaging Centre (CUBIC), Tygerberg Hospital, Cape Town using a Siemens Magnetom 3T Allegra MRI scanner (Erlangen, Germany). Infants were fed, swaddled in a blanket, and encouraged to sleep. A qualified nurse or pediatrician remained in the scanner room with the infant at all times, and a pulse oximeter monitored pulse and oxygenation throughout the scan. Given the challenges scanning neonates, a radiofrequency transmit/receive head coil was used with a wet clay inlay (40x40cm, 2cm thickness, standard sculpting clay) and voltage was decreased to optimize signal. Sagittal T2-weighted images were acquired using the following parameters: repetition time (TR) = 3500ms; echo time (TE) = 354 ms; FOV = 160 x 160 mm; in-plane resolution= 1.3 x 1.3 x 1.0mm, 128 slices. Sequence scan time was 5 minutes 41 seconds.

Neuroimaging processing

Images were first converted from DICOM to NIfTI format using the dcm2nii conversion tool. NIfTI images were then brain extracted using the FSL 5.0 brain extraction tool (BET) [1]. Each scan was visually checked following the initial BET and additional thresholds were applied to further improve the brain extraction.

Acquired structural T2-weighted images were processed using Statistical Parametric Mapping Software (SPM8) (www.fil.ion.ucl.ac.uk/spm/software/spm8) run in Matlab R2017B, using the University of North Carolina (UNC) custom infant T2 template in Montreal Neurological Institute (MNI) standard space [2]. Data from 95 infants (39 females, 56 males) were used to create this template and the associated tissue probability maps served as priors for segmentation. The UNC infant atlas has demonstrated improved performance when compared to manual segmentation as well as other comparable atlases [2-4]. In brief, the T2 images were registered to the UNC infant template and subsequently spatially normalised, where bias corrections and smoothing were applied as per the

SPM8 default settings. Images were segmented into grey matter, white matter, and cerebrospinal fluid using tissue priors as per the UNC infant template, and the resulting probabilistic maps were saved for further analysis [2]. During preprocessing, T2 images were modulated to preserve the volumetric information despite relative volumetric increases and decreases during image warping.

Neuroimaging data quality assessment

The normalized images and segmented grey matter maps were visually inspected by two researchers for segmentation accuracy and proper alignment to the template. Where there was disagreement over alignment quality, a third researcher reviewed the scans and made a final decision. Any images that failed normalization (n=4), or segmentation (n=13) were discarded. Masking and extraction were performed using the Masking toolbox [5]. Outlier detection was performed using the ENIGMA protocol (http://enigma.ini.usc.edu/protocols/imaging-protocols/) [6], to determine any values that were greater than 1.5 times the interquartile range. Any regions marked as a statistical outlier were re-inspected to evaluate segmentation.

Sensitivity analyses

Sensitivity analyses were performed (1) excluding imaging outliers (per the ENIGMA guidelines); (2) restricting analyses to children ≤28 days old; (3) restricting the group to only those children from one recruitment clinic, as this was closely correlated to HIV status; (4) including maternal prenatal alcohol use, smoking and depression, as these differed between the groups, and may be potential confounders; and (5) excluding children who tested positive for CMV at any point.

Table S1: Sociodemographic characteristics of children with neuroimaging versus those without in the Drakenstein Child Health Study

Variables	DCHS	Neonatal imaging versus no imaging		
	All children (n=1141)†	Imaging (n=146)	No imaging (n=995)	*P*
HIV exposure	247 (21.7%)	40 (27.4%)	207 (20.8%)	0.071
Male sex	586 (51.4%)	74 (50.7%)	512 (51.5%)	0.862
Site (Mbekweni)	632 (55.4%)	73 (50.0%)	559 (56.2%)	0.161
Monthly household income (ZAR)				
< R1000 (<~$75)	431 (37.8%)	49 (33.6%)	382 (38.4%)	
R1000-R5000 (~$75-375)	556 (48.7%)	75 (51.4%)	481 (48.3%)	
>R5000 (>~$375)	154 (13.5%)	22 (15.1%)	132 (13.3%)	0.514
Maternal education				
Primary	85 (7.5%)	7 (4.8%)	78 (7.8%)	
Secondary	609 (53.4%)	73 (50.0%)	536 (53.9%)	
Completed secondary	374 (32.8%)	58 (39.7%)	316 (31.8%)	
Any tertiary	73 (6.4%)	8 (5.5%)	65 (6.5%)	0.201
Maternal employment status (employed)	307 (26.9%)	40 (27.4%)	267 (26.8%)	0.886
Relationship status (married / cohabitating)	460 (40.4%)	63 (43.5)	397 (39.9)	0.416
Maternal age at birth, years	26.0 (22.3 - 31.1)	26.9 (22.0 – 31.6)	26.0 (22.3 – 31.0)	0.304
Gestational age at delivery, weeks	39 (38 – 40)	39 (38 – 40)	39 (37 – 40)	0.054
Birthweight, g	3025 (604)	3157 (462)	3005 (620)	0.025*
Maternal smoking during pregnancy				
Active	351 (32.2%)	45 (31.0%)	306 (32.4%)	
Passive	479 (43.9%)	61 (42.1%)	418 (44.2%)	
Non-smoker	261 (23.9%)	39 (26.9%)	222 (23.5%)	0.667
Maternal alcohol use during pregnancy	131 (13.2%)	25 (17.9%)	106 (12.4%)	0.077
Maternal depression	238 (23.9%)	44 (31.4%)	194 (22.6%)	0.023*
Maternal and child HIV variables (HEU children)				
Maternal HIV diagnosis timepoint				
Before pregnancy	174 (72.2%)	27 (71.1%)	147 (72.4%)	
During pregnancy	67 (27.8%)	11 (29.0%)	56 (27.6%)	0.864
Maternal CD4 cell count				
Median (range) (cells/mm^3)	411 (285 – 594)	423 (286 – 594)	408 (283 – 593)	0.925
<350 cells/mm^3	80 (40.2%)	13 (39.4%)	67 (40.4%)	
350-500 cells/mm^3	44 (22.1%)	8 (24.2%)	36 (21.7%)	
>500 cells/ mm^3	75 (37.7%)	12 (36.4%)	63 (38.0%)	0.948
Maternal Viral load (VL) in pregnancy				
Lower than detectable limit (<40 copies/mL)	93 (64.1%)	19 (73.1%)	74 (62.2%)	
VL detectable (40-1000 copies/mL)	30 (20.7%)	5 (19.2%)	25 (21.0%)	
Virally unsuppressed (>1000 copies/mL)	22 (15.2%)	2 (7.7%)	20 (16.8%)	0.450

Antiretroviral drug initiation				
Before conception	96 (40.0%)	15 (37.5%)	81 (40.5%)	
During pregnancy	144 (60.0%)	25 (62.5%)	119 (59.5%)	0.724
Antiretroviral regimen during pregnancy				
Monotherapy with AZT (zidovudine)	36 (15.0%)	5 (12.5%)	31 (15.6%)	
2 NRTIs + NNRTI (1st line non-EFV)	25 (10.4%)	3 (7.5%)	22 (11.0%)	
2 NRTIs + NNRTI (1st line EFV-containing)	166 (69.2%)	31 (77.5%)	135 (67.5%)	
2 NRTIs + PI (2nd / 3rd line)	13 (5.4%)	1 (2.5%)	12 (6.0%)	0.609
Infant prophylaxis				
NVP (nevirapine) alone	210 (87.1%)	35 (87.5%)	175 (87.1%)	
NVP + AZT	31 (12.9%)	5 (12.5%)	26 (12.9%)	0.940

Legend: Data are n/N (%), mean (SD) or median (IQR). Continuous variables were compared with unpaired t-tests or Mann-Whitney U tests; categorical variables were compared with Chi-squared. *p<0.05. †Full DCHS cohort excluding two children with HIV-infection. All HIV-related variables are cited out of the number of HIV-infected mothers with available data. Percentages are cited among those with non-missing values. Missing data: relationship status (n=1); maternal age (n=3); smoking (n=50); alcohol (n=146); depression (n=143); birthweight (n=11); gestational age (n=5); Maternal HIV diagnosis timepoint (n=6); CD4 cell count (n=48); Viral load (n=102); ART timepoint (n=7); ART regimen (n=7); Infant prophylaxis (n=6); Specific variables were assessed as follows: (i) Maternal smoking levels by urine cotinine, ≥500 ng/ml quantified active smoking, 10-500 mg/ml as passive smoking and <10ng/ml as non-smoking. (ii) Maternal alcohol use assessed and quantified using the Alcohol, Smoking and Substance Involvement Screening Test (ASSIST) and retrospectively collected data on moderate-severe alcohol use in pregnancy forming a dichotomous measure; (iii) Maternal depression in pregnancy measured using the Edinburgh postnatal depression scale (EPDS), a threshold of ≥13 was used for depression; (iv) The lowest maternal CD4 during pregnancy was used to reflect maternal immunosuppression in pregnancy; (v) Maternal viral load was categorised into <40 copies/ml as lower than the detectable limit, ≥40-<1000copies/ml as detectable and ≥1000copies/ml as unsuppressed. Where there was more than one result, the highest viral load during pregnancy was taken. Abbreviations: HEU = HIV-exposed and uninfected; HU = HIV-unexposed; VL = viral load; NVP = nevirapine; AZT = zidovudine, EFV = efavirenz; NRTI = nucleoside reverse transcriptase inhibitor; NNRTI = non-nucleoside reverse transcriptase inhibitor; PI = protease inhibitor

Table S2: Adjusted mean differences in grey matter volumes according to HIV exposure status excluding statistical outliers

Brain volumes	*N*		Mean (SD) volume (mm^3)		Minimally adjusted† coefficient (95% CI)	P-value	Effect size	Fully Adjusted‡ coefficient (95% CI)	P-value	Effect size
	HEU	HU	HEU	HU			Cohen's d (95% CI)			Cohen's d (95% CI)
Global volume										
Total grey matter	39	104	234,393 (14,983)	239,041 (11,898)	-4365 (-8510 to -220)	0.039*	-0.33 (-0.70 to 0.04)	-4305 (-8567 to -43.95)	0.048*	-0.33 (-0.70 to 0.04)
Subcortical regions										
Thalamus	38	102	3214 (106)	3214 (90)	5.22 (-30.06 to 40.49)	0.770	0.06 (-0.32 to 0.43)	5.94 (-30.12 to 42.00)	0.745	0.06 (-0.31 to 0.44)
Caudate	39	103	1786 (157)	1893 (153)	-105.54 (-161.48 to -49.59)	0.0003*§	-0.63 (-1.01 to -0.25)	-104.50 (-161.52 to -47.48)	0.0004*§	-0.62 (-1.00 to -0.25)
Putamen	37	101	2833 (70)	2843 (75)	-8.43 (-36.27 to 19.40)	0.550	-0.11 (-0.49 to 0.26)	-7.71 (-36.46 to 21.04)	0.597	-0.11 (-0.48 to 0.27)
Pallidum	38	101	714 (27)	713 (28)	1.64 (-8.51 to 11.78)	0.750	0.06 (-0.31 to 0.43)	1.09 (-9.35 to 11.52)	0.837	0.04 (-0.33 to 0.41)
Hippocampus	38	104	1582 (141)	1606 (123)	-13.76 (-57.37 to 29.85)	0.534	-0.11 (-0.48 to 0.26)	-13.43 (-58.19 to 31.33)	0.554	-0.10 (-0.48 to 0.27)
Amygdala	36	104	543 (23)	542 (21)	1.87 (-5.59 to 9.32)	0.621	0.09 (-0.29 to 0.47)	1.99 (-5.69 to 9.68)	0.608	0.09 (-0.29 to 0.47)

Legend: Subcortical volumes (mean of left and right hemispheres), mean differences (regression coefficients minimally and fully adjusted in multiple regression models), p-values and effect sizes for associations between brain volumes and HIV-exposure excluding outliers. Outliers were dropped bilaterally per region if either hemisphere was an outlier. Total intracranial volume outliers were dropped for all analyses. Effect sizes were calculated using Cohen's d with associated 95% confidence intervals. †Minimally adjusted models included child age at scan, child sex and intracranial volume. ‡Fully adjusted models included child age at scan, child sex, intracranial volume, maternal education, household income, and maternal age. Linear regression models where negative estimates indicate HIV exposure is associated with lower volumes in that region. *Uncorrected p<0·05. § p-values survive multiple comparison correction using the false discovery rate across subcortical regions. Abbreviations: HEU = HIV-exposed and uninfected; HU = HIV-unexposed; CI = Confidence Interval.

Table S3: Adjusted mean differences in grey matter volumes according to HIV exposure status restricted to children ≤28 days

Brain volumes	Mean (SD) volume (mm^3)		Minimally adjusted† coefficient (95% CI)	P-value	Effect size Cohen's d (95% CI)	Fully Adjusted‡ coefficient (95% CI)	P-value	Effect size Cohen's d (95% CI)
	HEU (n=34)	HU (n=87)						
Global volume								
Total grey matter	233969 (14815)	238812 (12086)	-4796 (-9322 to -270)	0.038*	-0.36 (-0.76 to 0.04)	-4998 (-9671 to -325)	0.036*	-0.38 (-0.78 to 0.02)
Subcortical regions								
Thalamus	3208 (112)	3220 (94)	-8.08 (-47.61 to 31.45)	0.686	-0.08 (-0.48 to 0.32)	-9.13 (-49.54 to 31.29)	0.655	-0.09 (-0.49 to 0.30)
Caudate	1799 (158)	1884 (155)	-83.89 (-146.70 to -21.09)	0.009*	-0.51 (-0.91 to -0.11)	-93.35 (-157.86 to -28.84)	0.005*§	-0.57 (-0.97 to -0.16)
Putamen	2837 (88)	2839 (84)	-3.48 (-36.87 to 29.92)	0.837	-0.04 (-0.44 to 0.36)	-0.75 (-35.53 to 34.04)	0.966	-0.01 (-0.41 to 0.39)
Pallidum	710 (34)	714 (29)	-4.74 (-16.51 to 7.03)	0.427	-0.16 (-0.55 to 0.24)	-4.38 (-16.76 to 8.01)	0.485	-0.14 (-0.54 to 0.25)
Hippocampus	1577 (145)	1611 (120)	-24.29 (-71.86 to 23.29)	0.314	-0.19 (-0.59 to 0.21)	-36.87 (-84.75 to 11.00)	0.130	-0.29 (-0.68 to 0.11)
Amygdala	537 (33)	543 (21)	-4.45 (-13.64 to 4.73)	0.339	-0.18 (-0.58 to 0.22)	-5.43 (-14.93 to 4.07)	0.260	-0.22 (-0.62 to 0.18)

Legend: Subcortical volumes (mean of left and right hemispheres), mean differences (regression coefficients minimally and fully adjusted in multiple regression models), p-values and effect sizes for associations between brain volumes and HIV-exposure restricted to children ≤28 days (n=121). Effect sizes were calculated using Cohen's d with associated 95% confidence intervals. †Minimally adjusted models included child age at scan, child sex and intracranial volume. ‡Fully adjusted models included child age at scan, child sex, intracranial volume, maternal education, household income, and maternal age. Linear regression models where negative estimates indicate HIV exposure is associated with lower volumes in that region. *Uncorrected $p<0{\cdot}05$. ¶p-values survive multiple comparison correction using the false discovery rate across subcortical regions. Abbreviations: HEU = HIV-exposed and uninfected; HU = HIV-unexposed; CI = Confidence Interval.

Table S4: Adjusted mean differences in grey matter volumes according to HIV exposure status restricted to Mbekweni clinic

Brain volumes	Mean (SD) volume (mm^3)		Minimally adjusted† coefficient (95% CI)	P-value	Effect size	Fully Adjusted‡ coefficient (95% CI)	P-value	Effect size
	HEU (n=39)	HU (n=34)			Cohen's d (95% CI)			Cohen's d (95% CI)
Global volume								
Total grey matter	233,675 (15,602)	238,679 (12,069)	-5730 (-10836 to -624)	0.028*	-0.40 (-0.86 to 0.07)	-5684 (-10941 to -427)	0.035*	-0.39 (-0.86 to 0.07)
Subcortical regions								
Thalamus	3204 (114)	3187 (98)	19.86 (-29.54 to 69.26)	0.425	0.19 (-0.27 to 0.65)	18.67 (-30.18 to 67.52)	0.448	0.18 (-0.28 to 0.64)
Caudate	1781 (154)	1840 (144)	-65.70 (-133.76 to 2.37)	0.058	-0.42 (-0.89 to 0.04)	-73.30 (-139.98 to -6.62)	0.032*	-0.47 (-0.94 to -0.00)
Putamen	2832 (86)	2839 (93)	-11.08 (-50.58 to 28.41)	0.577	-0.12 (-0.58 to 0.34)	-13.99 (-53.05 to 25.08)	0.477	-0.16 (-0.62 to 0.30)
Pallidum	711 (33)	713 (29)	-4.43 (-18.61 to 9.76)	0.536	-0.14 (-0.60 to 0.32)	-5.13 (-19.25 to 9.00)	0.471	-0.16 (-0.63 to 0.30)
Hippocampus	1564 (164)	1557 (132)	7.40 (-45.16 to 59.95)	0.780	0.05 (-0.41 to 0.51)	6.35 (-46.18 to 58.89)	0.810	0.04 (-0.42 to 0.50)
Amygdala	535 (32)	540 (23)	-5.24 (-16.78 to 6.30)	0.368	-0.18 (-0.65 to 0.28)	-5.32 (-17.24 to 6.60)	0.376	-0.19 (-0.65 to 0.27)

Legend: Subcortical volumes (mean of left and right hemispheres), mean differences (regression coefficients minimally and fully adjusted in multiple regression models), p-values and effect sizes for associations between brain volumes and HIV-exposure restricted to children attending Mbekweni clinic (n=73). Effect sizes were calculated using Cohen's d with associated 95% confidence intervals. †Minimally adjusted models included child age at scan, child sex and intracranial volume. ‡Fully adjusted models included child age at scan, child sex, intracranial volume, maternal education, household income, and maternal age. Linear regression models where negative estimates indicate HIV exposure is associated with lower volumes in that region. *Uncorrected $p<0{\cdot}05$. Abbreviations: HEU = HIV-exposed and uninfected; HU = HIV-unexposed; CI = Confidence Interval.

Table S5: Grey matter volumes according to HIV exposure status assessing the effect of maternal depression on the exposure-outcome relationship

Brain volumes	Minimally adjusted† coefficient (95% CI)	P-value	Effect size<br>Cohen's d (95% CI)	Fully Adjusted‡ coefficient (95% CI)	P-value	Effect size<br>Cohen's d (95% CI)
Global volume						
Total grey matter	-5388 (-9687 to -1089)	0.014*	-0.40 (-0.78 to -0.02)	-5032 (-9432 to -632)	0.025*	-0.37 (-0.75 to 0.01)
Subcortical regions						
Thalamus	14.33 (-26.15 to 54.82)	0.485	0.13 (-0.25 to 0.51)	17.07 (-24.58 to 58.71)	0.419	0.16 (-0.22 to 0.54)
Caudate	-91.05 (-150.53 to -31.56)	0.003*§	-0.54 (-0.92 to -0.16)	-87.39 (-147.29 to -27.49)	0.005*§	-0.52 (-0.90 to -0.14)
Putamen	-0.17 (-32.33 to 31.99)	0.992	-0.002 (-0.38 to 0.38)	0.12 (-33.11 to 33.35)	0.994	0.001 (-0.38 to 0.38)
Pallidum	0.10 (-11.55 to 11.75)	0.986	0.003 (-0.38 to 0.38)	0.77 (-11.26 to 12.81)	0.899	0.02 (-0.35 to 0.40)
Hippocampus	-16.56 (-62.92 to 29.81)	0.481	-0.12 (-0.50 to 0.26)	-12.96 (-60.63 to 34.71)	0.592	-0.09 (-0.47 to 0.29)
Amygdala	-1.46 (-9.90 to 6.98)	0.733	-0.06 (-0.44 to 0.32)	-0.24 (-8.92 to 8.44)	0.956	-0.01 (-0.39 to 0.37)

Legend: Mean differences (regression coefficients minimally and fully adjusted in multiple regression models), p-values and effect sizes for associations between brain volumes and HIV-exposure accounting for antenatal maternal depression. Antenatal maternal depression was measured using the Edinburgh Postnatal Depression Scale (EPDS) where a cut-off score of ≥13 was used as the threshold for depression. Depression was used as a proxy for maternal psychosocial illness; however, further work needs to be done to assess this potential mediator. Effect sizes were calculated using Cohen's d with associated 95% confidence intervals. †Minimally adjusted models included child age at scan, child sex, intracranial volume, and maternal depression during pregnancy. ‡Fully adjusted models included child age and child sex, maternal education, household income, maternal age, and maternal depression. Linear regression models where negative estimates indicate HIV exposure is associated with lower volumes in that region. *Uncorrected p<0·05. § p-values survive multiple comparison correction using the false discovery rate across subcortical regions. Abbreviation: CI = Confidence Interval.

Table S6: Grey matter volumes according to HIV exposure status assessing the effect of smoking on the exposure-outcome relationship

Brain volumes	Minimally adjusted† coefficient (95% CI)	P-value	Effect size Cohen's d (95% CI)	Fully Adjusted‡ coefficient (95% CI)	P-value	Effect size Cohen's d (95% CI)
Global volume						
Total grey matter	-5188 (-9338 to -1037)	0.015*	-0.39 (-0.75 to -0.02)	-5264 (-9509 to -1019)	0.015*	-0.39 (-0.76 to -0.02)
Subcortical regions						
Thalamus	6.09 (-32.98 to 45.17)	0.758	0.06 (-0.31 to 0.42)	8.75 (-31.46 to 48.97)	0.668	0.08 (-0.28 to 0.45)
Caudate	-90.79 (-149.47 to -32.10)	0.003*§	-0.54 (-0.91 to -0.17)	-83.51 (-142.40 to -24.61)	0.006*§	-0.50 (-0.87 to -0.13)
Putamen	-10.30 (-41.20 to 20.60)	0.511	-0.12 (-0.49 to 0.24)	-8.58 (-40.37 to 23.21)	0.594	-0.10 (-0.47 to 0.26)
Pallidum	-3.49 (-14.49 to 7.51)	0.532	-0.11 (-0.48 to 0.25)	-3.57 (-14.94 to 7.79)	0.535	-0.12 (-0.48 to 0.25)
Hippocampus	-26.54 (-72.21 to 19.12)	0.252	-0.19 (-0.55 to 0.18)	-27.52 (-74.63 to 19.58)	0.250	-0.20 (-0.56 to 0.17)
Amygdala	-3.46 (-11.83 to 4.90)	0.414	-0.14 (-0.51 to 0.22)	-3.33 (-12.02 to 5.36)	0.450	-0.14 (-0.50 to 0.23)

Legend: Mean differences (regression coefficients minimally and fully adjusted in multiple regression models), p-values and effect sizes for associations between brain volumes and HIV-exposure accounting for maternal smoking during pregnancy. Effect sizes were calculated using Cohen's d with associated 95% confidence intervals. †Minimally adjusted models included child age at scan, child sex, intracranial volume, and maternal smoking during pregnancy. ‡Fully adjusted models included child age and child sex, maternal education, household income, maternal age, and maternal smoking. Linear regression models where negative estimates indicate HIV exposure is associated with lower volumes in that region. *Uncorrected $p<0{\cdot}05$. § p-values survive multiple comparison correction using the false discovery rate across subcortical regions. Abbreviation: CI = Confidence Interval.

Table S7: Grey matter volumes according to HIV exposure status assessing the effect of alcohol on the exposure-outcome relationship

**Brain volumes**	**Minimally adjusted† coefficient (95% CI)**	**P-value**	**Effect size Cohen's d (95% CI)**	**Fully Adjusted‡ coefficient (95% CI)**	**P-value**	**Effect size Cohen's d (95% CI)**
Global volume						
Total grey matter	-4493 (-8772 to -214)	0.040*	-0.33 (-0.71 to 0.05)	-4272 (-8637 to 94)	0.055	-0.32 (-0.70 to 0.06)
Subcortical regions						
Thalamus	8.17 (-32.60 to 48.93)	0.693	0.08 (-0.30 to 0.45)	12.16 (-29.58 to 53.90)	0.565	0.11 (-0.27 to 0.49)
Caudate	-103.84 (-164.40 to -43.27)	0.0009*§	-0.61 (-1.00 to -0.22)	-102.23 (-163.69 to -40.77)	0.001*§	-0.60 (-0.99 to -0.22)
Putamen	-1.01 (-33.27 to 31.26)	0.951	-0.01 (-0.39 to 0.37)	0.67 (-32.59 to 33.94)	0.968	0.01 (-0.37 to 0.39)
Pallidum	-0.37 (-12.04 to 11.30)	0.950	-0.01 (-0.39 to 0.37)	-0.27 (-12.28 to 11.75)	0.965	-0.01 (-0.39 to 0.37)
Hippocampus	-20.86 (-67.79 to 26.07)	0.381	-0.15 (-0.53 to 0.23)	-19.94 (-68.39 to 28.51)	0.417	-0.14 (-0.52 to 0.24)
Amygdala	-2.98 (-11.60 to 5.65)	0.496	-0.12 (-0.50 to 0.26)	-2.11 (-11.00 to 6.78)	0.639	-0.09 (-0.46 to 0.29)

Legend: Mean differences (regression coefficients minimally and fully adjusted in multiple regression models), p-values and effect sizes for associations between brain volumes and HIV-exposure accounting for maternal alcohol during pregnancy. Effect sizes were calculated using Cohen's d with associated 95% confidence intervals. †Minimally adjusted models included child age at scan, child sex, intracranial volume, and maternal alcohol during pregnancy. ‡Fully adjusted models included child age and child sex, maternal education, household income, maternal age, and maternal alcohol use. Linear regression models where negative estimates indicate HIV exposure is associated with lower volumes in that region. *Uncorrected p<0·05. § p-values survive multiple comparison correction using the false discovery rate across subcortical regions. Abbreviation: CI = Confidence Interval.

Table S8: Adjusted mean differences in grey matter volumes according to HIV exposure status excluding CMV positive cases

Brain volumes	Mean (SD) volume (mm^3)		Minimally adjusted† coefficient (95% CI)	P-value	Effect size	Fully Adjusted‡ coefficient (95% CI)	P-value	Effect size
	HEU (n=37)	HU (n=72)			Cohen's d (95% CI)			Cohen's d (95% CI)
Global volume								
Total grey matter	234,308 (15517)	238,645 (11836)	-4619 (-8965 to -274)	0.037*	-0.34 (-0.74 to 0.06)	-5477 (-10,029 to -926)	0.019*	-0.40 (-0.80 to -0.00)
Subcortical regions								
Thalamus	3200 (114)	3197 (106)	5.19 (-37.15 to 47.53)	0.809	0.05 (-0.35 to 0.44)	10.32 (-34.00 to 54.65)	0.645	0.10 (-0.30 to 0.49)
Caudate	1785 (149)	1884 (143)	-98.13 (-156.51 to -39.74)	0.001*§	-0.62 (-1.02 to -0.21)	-114.70 (-173.95 to -55.44)	<0.001*§	-0.71 (-1.12 to -0.30)
Putamen	2828 (79)	2826 (84)	-0.42 (-32.70 to 31.86)	0.980	-0.01 (-0.40 to 0.39)	0.20 (-34.06 to 34.46)	0.991	0.00 (-0.39 to 0.40)
Pallidum	709 (32)	710 (29)	-1.44 (-13.16 to 10.28)	0.808	-0.05 (-0.44 to 0.35)	-1.70 (-14.08 to 10.67)	0.785	-0.06 (-0.45 to 0.34)
Hippocampus	1571 (160)	1597 (130)	-20.21 (-67.00 to 26.58)	0.394	-0.14 (-0.54 to 0.25)	-25.94 (-75.28 to 23.40)	0.299	-0.18 (-0.58 to 0.21)
Amygdala	535 (32)	542 (21)	-6.30 (-15.38 to 2.78)	0.172	-0.24 (-0.64 to 0.15)	-6.78 (-16.43 to 2.86)	0.166	-0.26 (-0.66 to 0.13)

Legend: Subcortical volumes (mean of left and right hemispheres), mean differences (regression coefficients minimally and fully adjusted in multiple regression models), p-values and effect sizes for associations between brain volumes and HIV-exposure excluding CMV positive cases and limiting to those with a negative CMV result (n=109). Effect sizes were calculated using Cohen's d with associated 95% confidence intervals. †Minimally adjusted models included child age at scan, child sex and intracranial volume. ‡Fully adjusted models included child age at scan, child sex, intracranial volume, maternal education, household income, and maternal age. Linear regression models where negative estimates indicate HIV exposure is associated with lower volumes in that region. *Uncorrected p<0·05. § p-values survive multiple comparison correction using the false discovery rate across subcortical regions. Abbreviations: HEU = HIV-exposed and uninfected; HU = HIV-unexposed; CMV = cytomegalovirus. Abbreviation: CI = Confidence Interval.

Table S9: Impact of maternal HIV disease severity (immunological compromise) on caudate and total grey matter volumes

		Caudate (left)				Caudate (right)				Total grey matter			
	N	**Mean (mm^3)**	**SD (mm^3)**	**Adjusted coefficient† (95% CI)**	**P-value**	**Mean (mm^3)**	**SD (mm^3)**	**Adjusted Coefficient† (95% CI)**	**P-value**	**Mean (mm^3)**	**SD (mm^3)**	**Adjusted Coefficient† (95% CI)**	**P-value**
HU group	106	1862	158	*Reference*	-	1911	176	*Reference*	-	238,676	12,127	*Reference*	-
CD4 >500	12	1814	204	-48.82 (-145.42 to 47.78)	0.319	1820	183	-92.03 (-191.44 to 7.37)	0.069	238,184	17,458	-641 (-7210 to 5929)	0.847
CD4 350-500	8	1816	176	-49.27 (-166.52 to 67.98)	0.407	1820	116	-102.21 (-222.86 to 18.45)	0.096	235,248	14,329	-4872 (-12845 to 3102)	0.229
CD4 <350	13	1805	121	-56.15 (-153.47 to 41.16)	0.256	1794	135	-98.91 (-199.05 to 1.24)	0.053	229,323	13,543	-8271 (-14889 to -1653)	0.015*

Legend: Mean differences (adjusted regression coefficients with 95% CIs) and p-values for associations between brain volumes and maternal CD4 cell count during pregnancy (categorized as CD4>500, 350-500 or <350). †Models were adjusted to include child age at scan, child sex and intracranial volume. Linear regression models where negative estimates indicate CD4 is associated with lower volumes in that region. *p<0·05. Abbreviations: HU = HIV-unexposed. Test for dose-response in total grey matter volumes using the likelihood ratio test, p=0.045. Abbreviation: CI = Confidence Interval.

Table S10: Impact of maternal ART regimen and timing of initiation on caudate and total grey matter volumes in the children who are HIV-exposed and uninfected

		Caudate (left)		Caudate (right)		Total grey matter	
	N	**Adjusted coefficient† (95% CI)**	**P-value**	**Adjusted coefficient† (95% CI)**	**P-value**	**Adjusted coefficient† (95% CI)**	**P-value**
ART alone							
AZT only	5	*Reference*		*Reference*		*Reference*	
1st line triple therapy	34	125.38 (-26.48 to 277.25)	0.103	24.04 (-124.37 to 172.44)	0.744	9632 (-2119 to 21382)	0.105
ART initiation							
Before conception	15	*Reference*		*Reference*		*Reference*	
During pregnancy	25	65.41 (-41.45 to 172.27)	0.222	43.17 (-55.69 to 142.03)	0.381	-1769 (-9917 to 6378)	0.662

Legend: Mean differences (adjusted regression coefficients with 95% CIs) and p-values for associations between brain volumes and maternal ART regimen and timing. ART regimen was categorized into zidovudine (AZT) versus 1st line regimens (the one child born to a mother on a second-line protease inhibitor containing regimen was excluded due to small group sizes). ART timing was dichotomized into initiation before conception versus during pregnancy. †Models were adjusted to include child age at scan, child sex and intracranial volume. Abbreviations: ART = antiretroviral therapy; AZT = zidovudine; CI = Confidence Interval.

Supplementary references

1. Smith SM. Fast robust automated brain extraction. Human brain mapping **2002**; 17(3): 143-55.
2. Shi F, Yap PT, Wu G, et al. Infant brain atlases from neonates to 1- and 2-year-olds. PLoS One **2011**; 6(4): e18746.
3. Brown CJ, Miller SP, Booth BG, et al. Structural network analysis of brain development in young preterm neonates. Neuroimage **2014**; 101: 667-80.
4. Tzarouchi LC, Drougia A, Zikou A, et al. Body growth and brain development in premature babies: an MRI study. Pediatr Radiol **2014**; 44(3): 297-304.
5. Ridgway GR, Omar R, Ourselin S, Hill DL, Warren JD, Fox NC. Issues with threshold masking in voxel-based morphometry of atrophied brains. Neuroimage **2009**; 44(1): 99-111.
6. ENIGMA. Structural image processing protocols. Available at: http://enigma.ini.usc.edu/protocols/imaging-protocols/. Accessed 12th May.
